# Back Pain in the Era of Opioid Restriction and Implications for Neurosurgeons Based on Qualitative Findings From a Rural State

**DOI:** 10.7759/cureus.57389

**Published:** 2024-04-01

**Authors:** Cara L Sedney, Uchenna Okakpu, Patricia Dekeseredy, Robin A Pollini, Joshua M Rosenow, Treah Haggerty

**Affiliations:** 1 Neurosurgery, West Virginia University, Morgantown, USA; 2 Medicine, West Virginia University, Morgantown, USA; 3 Behavioral Medicine and Psychiatry, Epidemiology and Biostatistics, Public Health, West Virginia University, Morgantown, USA; 4 Neurological Surgery, Northwestern University Feinberg School of Medicine, Chicago, USA; 5 Family Medicine, West Virginia University, Morgantown, USA

**Keywords:** non-surgical treatment, treating low back pain, rural areas, substance use disorder (sud), opioids use, chronic lower back pain, general neurosurgery

## Abstract

Historically, back pain has been an inciting complaint for the initiation of opioids. Aggressive marketing of opioids to treat back pain coupled with the initiation of pain being treated as “the fifth vital sign” contributed to the emerging opioid crisis in the USA. West Virginia (WV) has long been considered the epicenter of the crisis. In 2018, the WV legislature passed a bill that placed prescribing limits on opioids. Our group set out to investigate the impacts of opioid prescribing restrictions through a sequential, mixed methods study evaluating prescription trends and stakeholder experiences. These stakeholder experiences generated emergent themes regarding the evolution of the opioid crisis up to and beyond the implementation of the bill, which is of relevance to neurosurgeons and back pain treatment. This study explores those findings for a neurosurgical audience. This study consisted of open-ended, semi-structured interviews with a purposive sample of 50 physicians, pharmacists, and patients in WV. Interviews were recorded and transcribed verbatim. Content analysis was utilized as the methodological orientation. Five theoretical domains relevant to the treatment of back pain emerged, describing the prevalence of opioid use, barriers to access care, the importance of opioids for function in resource-poor rural areas, disconnected and siloed care, and patient views on the impacts of pain care gaps and solutions. Spinal pain care in rural WV is complex due to identified challenges. Care siloing factors in suboptimal spinal pain care. Future work should define, implement, and assess the real-world effectiveness of treatment paradigms for the full spectrum of surgical and non-surgical back pain complaints. Neurosurgeons should be present in this arena.

## Introduction

Back pain is a common condition, with approximately 80% of people experiencing low back pain during their lifetime [1]. It is estimated that between 12% and 14% of the adult population in the United States seek medical treatment for back pain in any given year [2], making it a common complaint for which medical treatment is sought. Neurosurgeons comprise part of the treatment team for patients with back pain due to the predominance of patients with spinal complaints, many of which present to the neurosurgeon for care [3]. Historically, back pain has been an inciting complaint for the initiation of opioids, even though the effectiveness of opioid therapy in chronic non-cancer pain is estimated at only 30% in some studies. While neurosurgeons are not high prescribers of chronic opioid pain medications, the predominance of spinal pain conditions as the impetus for initial exposure to opioids and their long-term use creates relevance to neurosurgeons and other practitioners treating degenerative spinal conditions as the opioid crisis evolves.

Kiang et al. reported an increase in opioid prescriptions from 2003 to 2017 and that “back problem” was the most common diagnostic indication necessitating opioid medication and resulting in the prescription of approximately 2.87 billion morphine milligram equivalents (roughly a quarter of all opioids prescribed) to this patient population during this time frame [4]. However, the efficacy of long-term opioid use for chronic low back pain remains controversial [5], while recognition of the harms of opioid prescribing in terms of addiction and overdose deaths has increased. These findings have resulted in various mitigation attempts to curb prescribing and a precipitous decrease in opioid prescribing on a national level.

West Virginia (WV) has long been considered the epicenter of the opioid crisis in the United States. Merino et al. identified that sociocultural factors, a depressed economy, insufficient education, and high rates of prescribed opioids in WV fueled the highest incidence of opioid misuse in the country [6]. To curb the misuse of prescription opioids, in 2018, the WV legislature passed SB (Senate Bill) 273, which placed prescribing limits on opioids [7]. The law placed limits on prescribing amounts for opioids but included exceptions for cancer patients and people already on long-term opioid therapy. Our group set out to investigate the impacts of SB 273 on opioid prescribing through a sequential, mixed-methods study evaluating prescription trends and stakeholder experiences.

The overarching study is a sequential, explanatory mixed methods study regarding the impact of SB 273 on prescription trends and unanticipated harms in WV. The first phase quantitatively analyzed WV’s prescription drug monitoring data [8]. The analysis showed that SB 273 did change overall prescription numbers. Still, the changes were seen in chronic opioid prescriptions rather than new opioid prescriptions, which were the focus of the law. Therefore, other influences on prescribing patterns were likely at play. The second phase included qualitative interviews with stakeholders, including prescribers, dispensers, patients using opioids, and people using diverted or illicit substances, to understand the quantitative findings. These stakeholder experiences generated emergent themes regarding the evolution of the opioid crisis up to and beyond the implementation of SB 273, which are of relevance to neurosurgeons and to back pain treatment in the era of opioid restriction. We thus seek to detail those findings here for a neurosurgical audience.

## Materials and methods

This qualitative study was guided by content analysis described by Hsieh et al. [9]. Content analysis is the “subjective interpretation of the content of text data through the systematic classification process of coding and identifying themes or patterns” [9].

Participants: A purposive sample of 50 stakeholders was recruited, including prescribers, pharmacists, and patients/people who used prescribed, diverted, or illicit substances. Inclusion criteria for the three groups were as follows.

Prescribers: Prescribing opioids was practiced in WV before and after the implementation of SB 273.

Pharmacists: They dispense opioids, practiced in WV before and after the implementation of SB 273.

Patients: Previously or currently taking opioids (including diverted or illicit opioids), lived in WV before and after implementation of SB 273 (primary care sample).

Recruitment for all three stakeholder groups was done through the West Virginia Practice-Based Research Network (WVPBRN) [10], which is a primary care sample; accordingly, patients were recruited from a primary care and not neurosurgical sample. A maximum variation strategy was employed to ensure equitable distribution of age, gender, urban/rural, ethnicity, and medical practice type (for prescribers and dispensers) and acute/chronic pain or drug use (for patients). Interviews were reviewed as data collection progressed, and sample subsets were modified iteratively as theme saturation was achieved. Potential participants were contacted by phone or email, and a paper consent form was mailed to them if they agreed to participate. Those who returned a signed consent form were contacted by phone to complete the interview, for which they received a $30 gift card. No participants withdrew from the study. All study protocols were approved by the West Virginia University Institutional Review Board (IRB #1908659237).

A semi-structured interview guide was developed to elicit information on the following topics. Background of medical condition (patients) or medical/pharmacy practice (prescribers and pharmacists), experience with opioids, influences on prescribing/availability of opioids over the years, impact of SB 273 on prescribing or access, and impact of SB 273 on care.

Interviews were conducted by PD, a qualitative research-trained female nurse, and lasted 30-60 minutes. Audio recordings of the interviews were transcribed professionally and reviewed for accuracy (PD). Any identifiers (i.e., proper names) were removed from the transcripts before being uploaded into NVivo 12 qualitative software for analysis [11].

**Figure 1:**
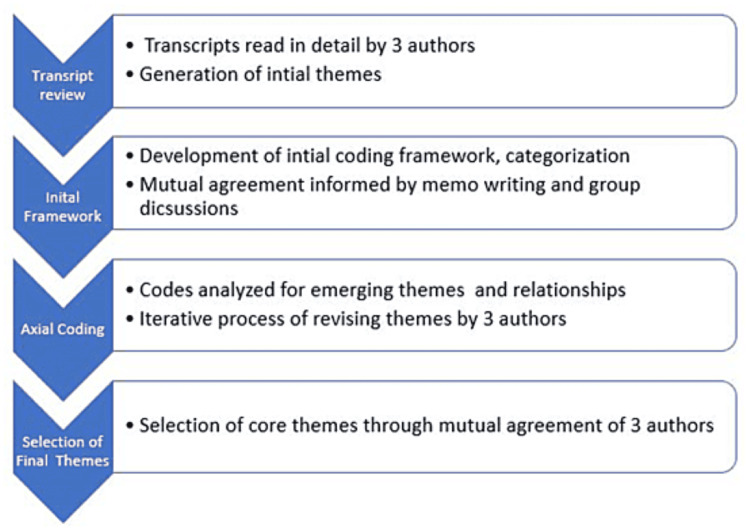
Coding Flow Diagram

This qualitative investigation is informed by a “mixed methods interpretivism” approach [12] to explain earlier quantitative information on opioid prescribing resulting from SB 273 [8], with the methodological orientation being content analysis. PD, CS, and TH independently read the transcripts for familiarization and developed a codebook. This initial coding was used to create a coding framework. The second step of the analysis was axial coding, in which emerging themes and concepts were explored for relationships by all three researchers. CS, PD, and TH utilized memo writing and group discussion to identify and expand codes. This was an iterative process in which all researchers merged and revised themes through dialogue and mutual agreement. This technique is in keeping with the techniques described by Sandelowski [13]. The themes were examined in relation to each other, including comparison and contrast of themes amongst various subgroups. An audit trail was maintained throughout data collection and analysis. A multidisciplinary research team improved methodological rigor (see Figure 1).

## Results

Prescriber participants included 20 physicians. Half were primary care physicians, and half were specialists, including surgeons, emergency room physicians, pain specialists, and physiatrists. Twelve were male, and eight were female. They were geographically distributed throughout the state of WV and had a range of 6-31 years in practice. Pharmacist participants included 10 pharmacists. They came from eight different counties across the state of WV and had a range of 5-30 years of practice in WV. Six were male, and four were female; they worked in various settings, such as community pharmacies, independent or chain pharmacies, academic pharmacies, etc. Patients included 13 female and seven male participants from 10 counties within WV. All patients had utilized opioids for either acute or chronic pain and/or used diverted/illicit substances during the implementation period for SB 273. While the original sampling strategy included discrete subgroups of patients with chronic pain and those with opioid use disorder, there was significant overlap between these groups in our sampling plan.

Specific participant characteristics are represented in Table 1.

**Table 1:** Participant Characteristics

	Male participants (n=25)	Female participants (n=25)	Range of years of practice	Number of West Virginia counties represented
Prescribers-Specialists	5	5	10-30	7
Prescribers-Primary care	7	3	6-30	7
Pharmacists	6	4	5-30	8
Patients	7	13	Not applicable	10

The preponderance of chronic spinal pain symptomatology among people using opioids

Participants widely recognized spinal pain as a predominant diagnosis for chronic pain for which opioids had been prescribed. Amongst prescribers and dispensers, anecdotes or examples of symptomatology relevant to opioids often involved back pain. Archetypal treatment of chronic pain using opioids was often described in terms of back pain.

You know, let’s say we have a, uh, a John Doe, who’s got intractable pain because he had a severe accident because he was a pipe liner and, you know, he’s got severe back pain and what gives him relief is, you know, um, Norco three times a day. And it’s legitimate and, you know, we tried surgery, nothing else helps him, you know… (Pharmacist 02)

Furthermore, amongst the 20 patients, all but one patient described back pain as part of their symptomatology - either as their onset of pain or exposure to opioids or as an exacerbating issue.

I had, had a back surgery in ’05 when I was in nursing school. It was a herniated disk that they went in and they shaved off what was getting the nerve. I worked for six years. I had, still, I had residual pain and stuff and a lot of that was from scar tissue but it wasn’t pushing on a nerve to where it was continuous pain. Then, in January 2011, that’s when I got hurt and I was on Workman’s Comp for 18 months, and in that time, I had two surgeries. (Patient 09)

Compounding the prevalence of back pain symptoms as driving the use of opioids for chronic pain, the patients acknowledged the limitations of all forms of treatment for back pain. Some of the participants had surgery for their spinal complaints with persisting pain after the surgery; others were prescribed opioids because surgery was not an option for them. Some patients related that treatments such as surgery were initially successful but had continued problems. Many were aware of their diagnoses, which they described as radiculopathy, stenosis, degenerative disc disease, and failed back surgery syndrome. Many detailed back injuries related to motor vehicle accidents or work-related injuries as inciting events for either the onset or worsening of pain.

The preponderance of back pain symptomatology as a reason for chronic opioid treatment in this group led to a significant effect on patients with back pain leading up to and after the implementation of SB 273 and its subsequent resulting care gaps for this particular group of patients [14].

Barriers to care of back pain in rural areas

All participant groups discussed barriers to pain care in rural areas, which often drove the need for opioids. These were relevant to spine care and included access issues related to rurality, geography, and insurance, making care more complex and less feasible for many patients in back pain treatment. Stakeholders noted this to be a historical problem, which drove the choice to utilize opioid pain medications to treat pain instead of other methods. However, it was also pertinent more recently as the barriers to pain care become more pronounced: local, primary care providers became less willing or able to prescribe opioid pain medications for chronic pain due to SB 273, Centers for Disease Control and Prevention guidelines, or other influences. All stakeholder groups prominently mentioned the lack of insurance coverage for conservative treatments such as physical therapy; specifically, physical therapy was much more financially inaccessible than opioid prescriptions based on most insurance coverage. These barriers were seen to drive the use of opioids for chronic pain.

One of the things is that we need this-- the insurance companies…need to start reimbursing for physical therapy, occupational therapy, and other modalities better than they currently are because at this point, it is cheaper for them to get a prescription of Oxycodone than to go to the physical therapist for one visit. (Specialist 10)

Similarly, some prescribers and dispensers mentioned the lack of evidence-based, complementary treatments for back pain, such as acupuncture and aqua therapy. Specifically, they contrasted its availability in rural WV with what was described by their colleagues in more urban locations. Care access disparities based on insurance type were also described.

It’s like the insurance company wouldn’t approve my surgeries here in Charleston, I went through four months where they forced me to take pain pills to travel 100 miles to get my surgery. (Patient 04)

As mentioned by the patient above, the long distance required to travel for specialized pain care, either surgical or non-surgical, was frequently mentioned by patients, prescribers, and dispensers. This became particularly prominent as local opioid prescribing decreased, and patients were required to go to pain clinics up to several hours away to access pain care. The cost of gas, the discomfort of traveling on roads in poor conditions, inclement weather, the inability to take time off work, or the inability to drive oneself while on opioid medication were seen as barriers to care.

My son-in-law’s sitting out there in the car. I’ll take one [opioid pill] before I ride down on this West Virginia terrible road. Or I’ll be going out of my mind by the time I get home… It’ll take us about two hours, following a black snake or a drunk cow [reference to winding mountain roads]. (Patient 14)

These became especially critical as prescribers of opioids became stricter with opioid refill protocols, and the intervals between required visits for chronic opioids decreased in accordance with guidance such as SB 273. Other infrastructure-related barriers included a lack of cell service or internet, which became relevant during the COVID-19 pandemic as providers offered telemedicine services. These barriers to care were exacerbated by opioid restriction measures such as SB 273 as fewer local physicians were willing to prescribe opioids, and patients were shuffled to specialty providers farther away, which further exacerbated care access for patients with back pain.

The perceived importance of opioids in maintaining functionality in rural, resource-poor areas amongst those with back pain

All stakeholder groups discussed opioids as playing some role in helping patients with primarily physical jobs such as mining or construction to maintain functionality and support their families in settings where other, less physically demanding employment was unavailable. Patients and providers described spinal injuries from such employment, such as falls from telephone poles and electrocution, with opioids being required after injury to maintain functionality.

Several prescribers and dispensers discussed their observations that some patients did well on long-term, stable-dose opioids, contrary to published evidence regarding the efficacy of opioids for chronic non-cancer pain.

What the-what the clinical trials say and what, at least what I experienced from a-in a day to day practice standpoint are two very different things. You know, because patients historically say that they do get some relief, and it does improve their quality of life, and it does make a difference, uh, versus, you know, most of the literature that would say that it doesn’t make a difference, and that actually makes their quality of life worse. (Primary Care 06)

The importance of opioids for maintaining functionality in the setting of spinal pain, either at work or in activities of daily living, was detailed by many patients.

When I was getting them regularly, I had a full-time job, my own place, I owned half of a business. I could function better. Now, I’m just like a lump on a log. (Patient 07)

Several patients detailed previous attempts to taper their opioids, which were unsuccessful because it significantly impaired their ability to function. These patients were distressed at the possibility of having opioids forcibly tapered in the future if further changes happened and wondered what the alternative would need to be for them to maintain their functionality, particularly as other care access issues were exacerbated, as detailed above.

If you’re going to restrict its usage, then if they take the opioid way and not give me another something to kill my pain, then what do I do? Do I go to medical marijuana? Do I start drinking alcohol? Do I turn into an alcoholic or something to kill the pain? Do I get so fed up I commit suicide? What do I do? Do I lay there and suffer? (Patient 14)

Medical specialty siloing and disconnect in pain care

Medical specialty siloing in chronic pain care was a prominent theme, with pharmacists and prescribers often at odds over their perception of indications and effectiveness of opioids. Prescribers often saw pharmacists as being hostile or punitive, particularly after the implementation of SB 273.

They’d call you and they’d refuse to fill your prescription. They’d say it was illegal and it violates the law and you’d feel bad for even asking them to. You get one or two calls from a pharmacist like that, you change your prescribing pattern pretty quickly. (Primary Care 02)

This dynamic contributed to prescribers’ fear of disciplinary action, which our group has previously detailed [14]; prescribers suspected pharmacists of unjustifiably reporting physicians to the state’s Board of Pharmacy or the US (United States) Drug Enforcement Administration. However, the dispensers, in turn, felt that physicians were not appropriately counseling or assessing their patients and that physicians were driven by the financial benefits of seeing such patients.

*I feel like a lot of doctors or prescribers that we’ve seen over the years, it’s just a money thing where they’re just writing to be writing. Some patients don’t really have an understanding as to how that can affect them. Some of our patients maybe not know how addictive medication may be. (Pharmacist 08*)

Similarly, siloing amongst specialists and primary care providers over whose purview pain care belonged contributed to treatment gaps in pain care. This was particularly exacerbated as opioid prescribing decreased leading up to and during the implementation of SB 273 as specialty pain care was explicitly recommended in the legislation but was not made more available or accessible. Pain specialists noted that SB 273 and other measures to decrease opioid prescribing drove large patient volumes to their practices, which were impossible to accommodate, mainly as other pain clinics shut down or prescribing physicians retired. Non-pain specialists, including surgeons, did not feel that addressing pain, except in very limited situations (such as the immediate post-operative period), was within their purview. Thus, they instructed patients to see primary care physicians for chronic pain. 

And so, you know, I’m not a primary care physician who’s going to give somebody, you know, a-a low dose narcotic for their back pain every month. (Specialist 02)

However, primary care physicians noted both fear of liability or disciplinary action from prescribing chronic opioids and also logistical limitations as increasing documentation and diagnostic requirements, such as those imposed by SB 273, made such care untenable in already overloaded primary care practices. This resulted in patients being shuffled from provider to provider and ultimately impeded their chronic pain care.

… talking about creation of-of a treatment plan [SB 273 requirement], proving that they have chronic pain, that would be appropriate for opioids. That is extremely hard to do… - time-consuming, to do- honestly, doesn’t really fit into the way our healthcare system is set up. You know, our healthcare system as a whole is largely- you got to see a ton of patients, uh, in order to generate enough revenue to keep the lights on. You know, and so it’s frustrating for me when they start pushing these significant sort of quality control issues, but still want you to work in a payment system that is based on quantity. You know, and so you’re wanting this in-depth history, this in-in depth, this investigation of what their pain is, what’s been done, MRIs, yada yada, yada, yada, like all of this stuff, but there’s barely any time to do it. And when you try to do it, insurance companies almost always will deny whatever request you have. (Primary Care 06)

Even as primary care physicians noted that chronic pain management in a primary care setting was less feasible, physicians acknowledged that pain specialists were often ill-equipped or unwilling to specifically manage oral pain medication in favor of procedural interventions such as spinal injections. The comprehensive management of pain was seen as sorely needed by primary care physicians and was of utmost value to them and their patients.

I think the highest paid physician, [laughs] honestly, should be the person who’s willing to take on their pain patients. Because that person, you know, is gonna help me more as a primary care doctor than the neurosurgeon, the orthopedic surgeon or whoever else it is that’s, you know, making a million dollars a year or whatever, you know? (Primary Care 06)

Patient views on impacts of pain care gaps and solutions

Care siloing and conflict between physicians and pharmacists or between primary care physicians and specialists were seen by patients as part of a broader social movement, depriving them of the needed care in many cases. Patients who were able to maintain their chronic opioid prescriptions felt fortunate to have done so. Those who were unable to continue chronic opioid prescriptions described forced tapering or inability to meet changing requirements by physicians to be on controlled medications. 

He did after seven years. He just completely dropped me and didn’t give me a no-wane-down dose, no nothing. Coming off of them was pretty hard. (Patient 10)

Several patients who were forcibly tapered transitioned to illicit substances precisely to control pain and maintain functionality rather than for purposes of recreational use.

They either suffer because they’re in too much pain or they have to buy something off the street... I couldn’t go to a doctor and be like, “My knee hurts, I need something for it,” because they wouldn’t do anything for that. They’re not going to give me any kind of opiate or anything. Because of that, I started buying Lortab and oxycodone again to try to continue working until the point that it was taking all my money that I was making, working to buy pills to work. If there was some way to work out where people that really had injuries to the body and stuff to get help, then I think it would reduce a lot of all of this street drugs use and everything. (Patient 03)

Other patients noted that their need for illicit substances drove increasing dependence, chaotic use, and addiction. Even short-term care gaps at times resulted in a transition to illicit substances.

At one point I was in a lot of pain and I didn’t have my medications due to a lapse in appointment times when I was done with my prescription. This guy, this dealer just said, “This is basically the same thing, you should try this.” (Patient 05)

Worsened functionality amongst patients who had a forced taper was significant, in both personal and professional roles. Some patients who had undergone a forced taper of opioids felt that there were no other comparable solutions for them and desired reinstituting opioid therapy. However, finding such care was seen as an insurmountable barrier. Other patients desired more individualized and personalized care for their back pain - whether or not that treatment included opioids.

I’m trying to find good doctors for me, that not necessarily just give me things for pain, but just to listen to me and understand what I have to go through every day. It’s hard. (Patient 02)

Patients considered balancing the needs of patients in pain with the need to minimize the harms of addiction to be a critically important issue moving forward, although they acknowledged that solutions to this difficult problem would not be easily operationalized.

## Discussion

Chronic low back pain management remains a challenge for patients and the clinicians who treat it [14]. These clinicians range from chiropractors and physical therapists to primary care providers, anesthesiologists, pain specialists, physiatrists, mental health professionals, orthopedic surgeons, and neurosurgeons. Patients suffering from back pain continue to experience a lower quality of life, especially if they have previously undergone and failed back surgery [15]. It has been reported that more than a quarter of US adults experience acute low back pain within three months and that 10% of patients develop chronic low back pain [16]. Because of changing legal, cultural, and economic forces within the healthcare environment amid the opioid crisis, the care of such patients is increasingly complex for primary care providers [17], resulting in unintended consequences for patients [14].

Previously noted trends in opioid prescriptions for back pain have demonstrated that patients of lower socioeconomic status were more likely to receive guideline-noncompliant care (prescriptions of opioids alone) for back pain than those of higher socioeconomic status [18]. Rural stakeholders in this report felt that opioids were their only option in many cases. The lack of other available treatments may compound this viewpoint: a 2020 study demonstrated that patients who completed physical therapy were less likely to receive an opioid prescription [19]; however, all of our stakeholder groups mentioned the inaccessibility of such services in rural areas.

Accessibility of “conservative treatments” for back pain is known to be diminished in rural areas, and this impacts treatment decisions and outcomes, according to our stakeholders. These included physical therapy and more alternative therapies with various levels of supporting evidence, such as acupuncture or cognitive behavioral therapy, etc. Such alternative therapies have been successfully implemented in rural areas with resulting improvements in pain outcomes [20] but are not widespread. The reasons for this dearth of conservative, evidence-based treatments appear to be multifactorial. In an investigation of the provision of physiotherapy services in rural locations, various factors such as the population size to support private practice and the presence of other healthcare resources such as hospitals and rehabilitation units influenced the availability of physical therapy in rural areas [21]. Regardless of the reason, this lack seems to relate to the increased dependence on opioid therapy for back pain.

Recognition of the adverse effects of opioids and the subsequent public and political outcry surrounding the opioid crisis has resulted in numerous mitigation attempts on the provision of prescription opioids, including updated guidelines, restrictive prescribing laws, and insurance coverage restrictions. This has resulted in a sea-change of opioid availability, including amongst cancer patients and other patients who experience benefits from opioids, whose care was intended to be considered separately by most of the newer guidelines and regulations [14]. We have previously reported on the unintended consequences of such mitigation attempts, with prescribers' fear of disciplinary action affecting their prescribing practices, and with the possible unintended consequence of care gaps in pain care and transition to illicit substances amongst patients with chronic pain [14]. When investigating broader stakeholder viewpoints on these care gaps, it becomes evident that significant care siloing concerning pain care is present. The lack of other efficacious treatments for pain magnifies such effects and leaves a potential role for neurosurgeons as care providers for people with back pain. This siloing is exacerbated in rural settings where the multidisciplinary providers are not geographically concentrated, leading to poor physical access by patients with limited transportation resources and poor communication among the providers who rarely interact in a shared care setting. Expanding multidisciplinary care or developing communication strategies or mechanisms to facilitate direct communication between care providers, including between healthcare systems, may improve such siloing.

Based upon the perceptions of opioid prescription stakeholders examined in this work, several conclusions regarding the neurosurgical role in spine-related pain care can be drawn and recommendations made. Spine-related pain complaints such as back pain are significant drivers of opioid prescribing - either appropriately or inappropriately - and this may be exacerbated by a lack of available evidence-based care, particularly in underserved and rural areas. Expanding the capability and format of treatment methods to reach less accessible patient populations is key to minimizing the need for opioid treatment. Telemedicine, app-based care, and other innovative techniques may assist in making such care more widely available and have demonstrated efficacy in rural populations [22]. For example, mindfulness is an evidence-based pain intervention that is largely unavailable to rural populations given the lack of pain psychologists, but for which several mobile interventions have been devised [23]. Additionally, experience with telephysical therapy has been improved due to the COVID-19 pandemic [24]. One recently reported care model for non-operative back pain includes a partnership of a rural nurse practitioner and an urban physical therapist providing care via telehealth [25]. To maximize the reach and sustainability of such measures, equitable insurance coverage should be available for the full spectrum of pain treatments, particularly as insurance initiatives have been shown to impact pain care access [26].

Care siloing is detrimental to care, and care models that decrease treatment gaps should be explored, particularly as chronic pain management becomes more complex, more regulated, and less feasible for primary care providers. This may involve neurosurgeons treating non-operative spinal pain or closely collaborating with non-operative providers to fulfill the need for non-operative spinal pain treatment, along with adequate reimbursement and legal protections for physicians treating chronic pain. Interestingly, the treatment of pain has been embraced within the functional neurosurgical community but less so in the spinal surgery community, despite degenerative spinal procedures being primarily pain procedures. Incorporating surgical and non-surgical care has previously been reported with good results in other neurosurgical pain conditions such as trigeminal neuralgia [27]. Furthermore, procedures specifically for treating chronic pain may be adapted and expanded to the purview of neurosurgeons [28]. Neurosurgeons already actively treat both acute and chronic pain with a variety of procedures, such as structural spinal surgery, cranial and peripheral nerve decompression, central nervous system ablative procedures, and neuromodulation. However, patients continue to have access limitations to these evidence-based procedures due to deficits in knowledge of their existence among both patients and primary care physicians, in the availability of surgeons who have the requisite expertise (especially in rural areas), and in insurance coverage (which is often the largest and most difficult hurdle to overcome). In many cases, surgical intervention may aid in the goal of decreasing opioids. The value of neurosurgical procedures should be considered when considering pain care as a whole.

There is, furthermore, precedent at an organizational level for the involvement of surgeons in developing capacity for essential non-surgical care services where a critical need is recognized. The American Orthopaedic Association initiated the “Own the Bone” program in 2005 to assess and improve the prevention of secondary fractures in patients who sustain fragility fractures [29]. This was done because osteoporosis was a significant public health issue, and surgeons were seen as critical actors in the solution [30]. These non-surgical interventions include counseling, bone density testing, and prevention efforts, including exercise, smoking cessation, and pharmaceutical interventions to treat osteoporosis, often by non-surgical providers within or affiliated with orthopedic centers. The need for similar interventions regarding the treatment of chronic back pain, which stretch the clinical capabilities of surgeons to fill critical gaps in care amongst patients with chronic spinal pain conditions, must be considered.

This qualitative study examines the views of opioid prescription stakeholders in WV and, as such, has some limitations. Because WV is a rural and underserved state, combined with historically high rates of opioid prescribing and illicit substance use, the conclusions drawn herein may not apply to other populations with different societal forces and prescribing trends. However, the predominance of back pain and the use of opioids for treatment due to access issues, care siloing, and poor availability of evidence-based pain care are broadly applicable to neurosurgeons treating painful spinal conditions and currently changing prescribing patterns of opioids seem to create a gap in spinal pain care that neurosurgeons may have a role in filling.

## Conclusions

Just as care siloing can be detrimental to patient health, policy siloing can also be. Well-intended initiatives to reduce opioid overprescribing must be done in conjunction with efforts to expand access to the full range of pain care. Doing the former without the latter leaves patients and their primary care physicians stranded without viable solutions to a critical problem, often leading patients to take matters into their own hands in ways that are not always legal or beneficial.

This study defines spinal pain complaints as a significant perpetrator of the opioid crisis and denotes implications for the treatment of back pain in the midst of decreasing opioid prescribing. Care siloing is an important factor in suboptimal spinal pain care. Future work should define, implement, and assess the real-world effectiveness of treatment paradigms for the full spectrum of surgical and non-surgical back pain complaints, and neurosurgeons should be present in this arena.
